# Synchronous lateral lymph node metastases from papillary and follicular thyroid carcinoma: case report and review of the literature

**DOI:** 10.1186/s13044-022-00120-w

**Published:** 2022-02-04

**Authors:** Adam Stenman, Magnus Kjellman, Jan Zedenius, C. Christofer Juhlin

**Affiliations:** 1 Department of Molecular Medicine and Surgery, Karolinska Institutet, Stockholm, Sweden; 2 Department of Breast, Endocrine Tumors and Sarcoma, Karolinska University Hospital, Stockholm, Sweden; 3 Department of Oncology-Pathology, Karolinska Institutet, 171 64 Solna, Sweden; 4 Department of Pathology and Cancer Diagnostics, Karolinska University Hospital, Stockholm, Sweden

**Keywords:** Papillary thyroid carcinoma, Follicular thyroid carcinoma, Lymph node metastasis, NRAS, BRAF, TERT, Prognosis

## Abstract

**Background:**

Follicular thyroid carcinomas (FTCs) rarely metastasize to regional lymph nodes, and descriptions of synchronous lateral lymph node metastases of FTC and papillary thyroid carcinoma (PTC) are lacking.

**Case Presentation:**

We describe a 43-year-old female with a preoperative cytology indicating a right-sided PTC with lateral lymph node metastases. She underwent a total thyroidectomy and central and lateral lymph node dissection, and histopathology confirmed a multifocal tall cell variant PTC together with a 12 mm minimally invasive FTC in the ipsilateral lobe. While the central compartment demonstrated metastatic PTC, the lateral compartment contained PTC metastases alongside a 15 mm large follicular-patterned mass in a separate lymph node. As the cells lacked PTC associated nuclear changes, the possibility of a lateral lymph node metastasis of FTC was considered, with the possibility of ectopic thyroid tissue as a differential diagnosis. By utilizing next-generation sequencing, a Q61R *NRAS* mutation was pinpointed, thus proving the tissue as tumorous. The patient underwent radioiodine treatment and is currently monitored following a negative whole-body scan.

**Conclusions:**

This is probably the first case report of a patient with co-existing lateral lymph node PTC and FTC metastases. Consulting previous publications, there is currently a gap of knowledge in terms of how patients with regional FTC metastases should be followed-up and treated, especially when co-occurring with spread high-risk PTC subtypes. Moreover, what guides a seemingly indolent FTC to spread via the lymphatic system remains to be defined from a molecular standpoint.

## Background

Treatment guidelines for well-differentiated thyroid cancer (WDTC) are streamlined, with the goal to remove primary tumor tissue and clinically significant lymph node metastases via surgery, thus reducing the risk of disease recurrence and metastatic spread [1]. Current guidelines for disseminated cases are heavily focused on the presence of a single tumor type, and little is known regarding the prognosis for cases with two separate types of WDTCs, especially when synchronously metastasized to the lateral neck. While papillary thyroid carcinomas (PTCs) often spread to regional lymph nodes, metastasized follicular thyroid carcinomas (FTCs) spread hematogenously, and only occasionally metastasize to the central or lateral neck compartment [2]. However, there are several reports of cervical lymph nodes containing benign ectopic thyroid tissue, most often localized to the central compartment [3]. Indeed, ectopic thyroid tissue is unusual in the lateral neck region, and metastasis from a thyroid tumor should almost always be ruled out clinically before a diagnosis of aberrant thyroid tissue is considered in this location [3–5]. In this case report, we describe a patient with a synchronous PTC and FTC with ipsilateral neck lymph node metastases of both tumor types, and discuss the diagnostic work-up, the therapeutic considerations and the previous literature of this unique manifestation.

## Case Presentation

The patient was a healthy 43-year-old female without previous history of thyroid illness and no family history suggestive of thyroid-related disorders. She had no prescribed medications at the time of presentation. In 2021, she noticed a swelling at the right side of the neck. Ultrasound revealed a 2.9 × 2.3 × 2.1 cm nodule in the right thyroid lobe as well as an enlarged right-sided lymph node in region IV. The subsequent fine-needle aspiration cytology (FNAC) examination of the primary lesion showed epithelial cells with nuclear grooves and pseudo-inclusions, consistent with PTC (Bethesda VI). FNAC from the lateral lymph node displayed epithelial cells with nuclear grooves and pseudo-inclusions, positive for TTF-1, consistent with metastatic PTC. The patient was planned for a total thyroidectomy with central node resection (region VI) along with a right-sided region II-IV modified neck dissection.

**Fig. 1:**
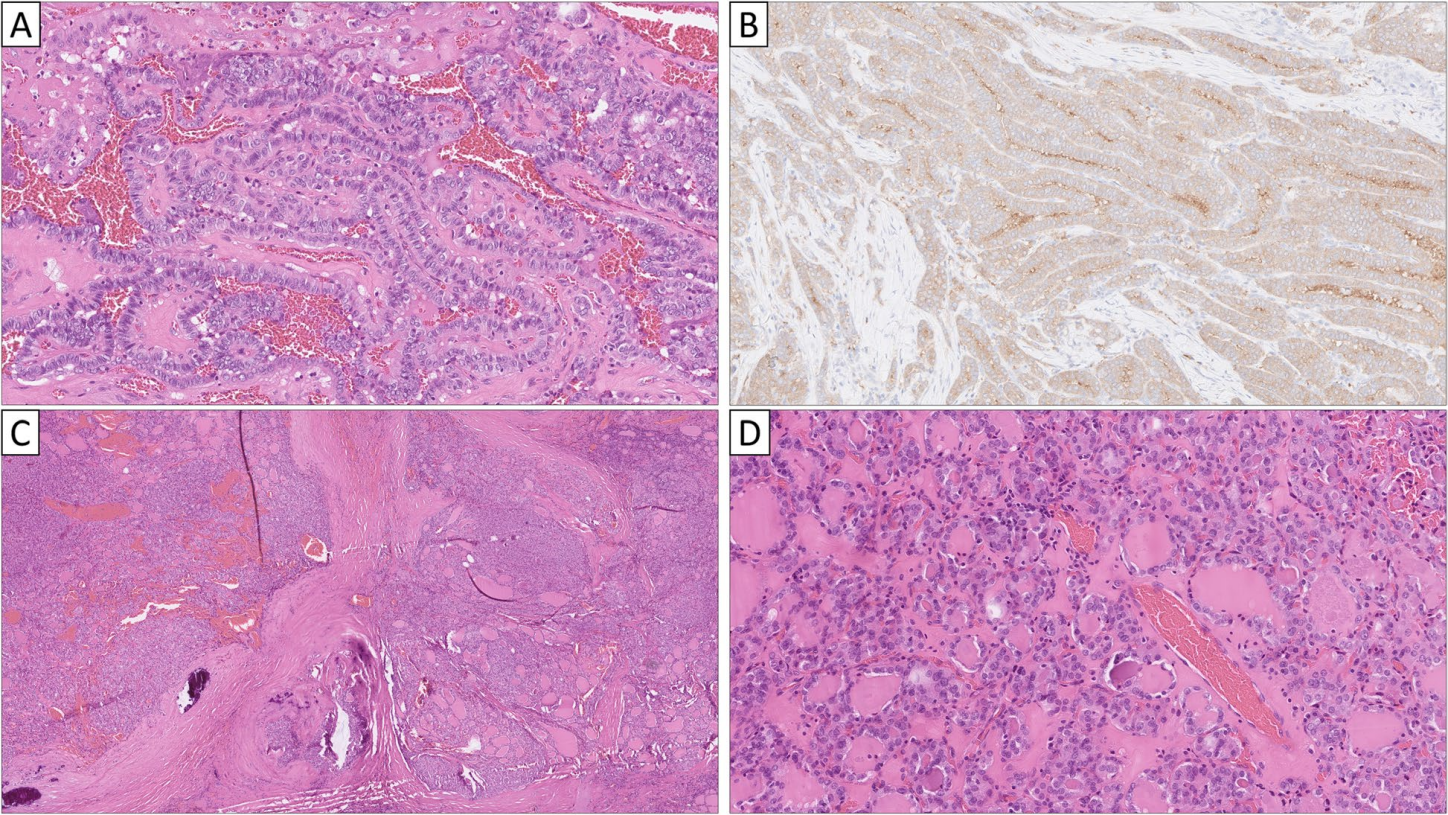
Histological and immunohistochemical attributes of the synchronous papillary (PTC) and follicular thyroid carcinoma (FTC). All stains are hematoxylin–eosin unless otherwise specified. **A** The largest focus of the multifocal tall cell variant PTC is shown. Note the eosinophilic, taller-than-wide tumor cells and the “tram-track” appearance of tumor nuclei. **B** This lesion was positive for BRAF1, the V600 mutation specific antibody. **C** The 12 mm minimally invasive FTC (miFTC) was characterized by multiple areas with capsular invasion, but lacking angio-invasive features. **D** High-power magnification of the miFTC reveals monomorphic nuclei lacking PTC related changes

The histological examination of the thyroid gland revealed a right-sided, multifocal tumor with predominant papillary growth, with the largest focus measuring 30 mm (Fig. 1A). The majority of tumor cells exhibited an eosinophilic cytoplasm with cells three times taller than wide, and tumor nuclei were enlarged, with crowding, nuclear folds and pseudo-inclusions. The tumor was positive for the V600 mutation-specific BRAF antibody (BRAF1) (Fig. 1B). Approximately 50% of tumor cells showed thyroglobulin immunoreactivity, and the Ki-67 proliferation index was 5.6%. No vascular invasion or extrathyroidal extension was noted. The final diagnosis was tall cell variant PTC (TCV-PTC). Intriguingly, a 12 mm follicular thyroid tumor was observed in the ipsilateral lobe containing areas with capsular invasion, and the lesion was diagnosed as a minimally invasive FTC (miFTC) (Fig. 1C-1D). This lesion was negative for BRAF1, and the Ki-67 index was 3%. Moreover, a 6 mm follicular hyperplastic nodule was seen, as well as a 3 mm non-invasive follicular thyroid neoplasm with papillary-like nuclear features (NIFTP) (data not shown).

**Fig. 2:**
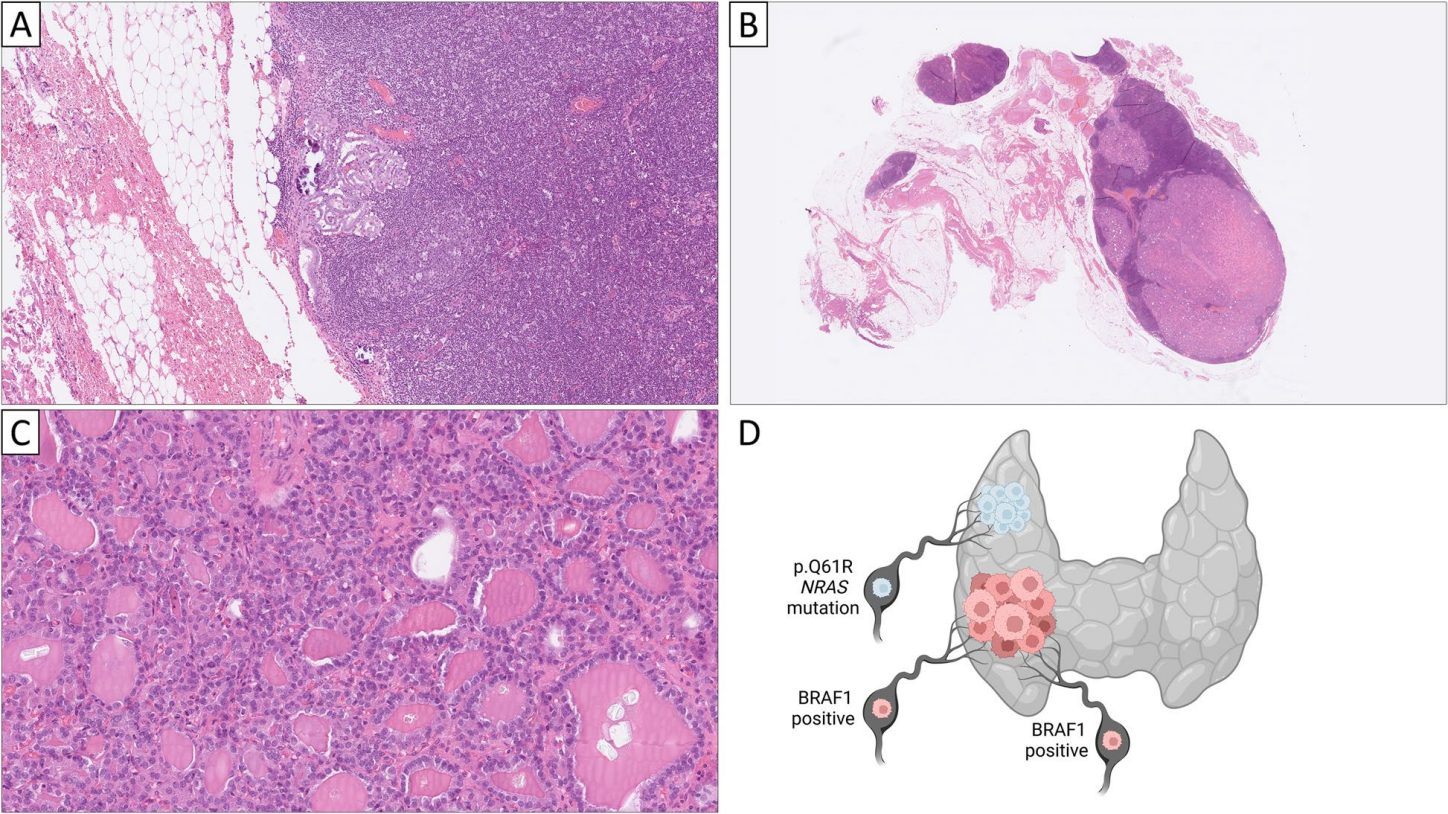
Morphological and genetic phenotypes of the lateral lymph node metastases. All stains are hematoxylin–eosin. **A** Approximately 1 mm large subcapsular deposit of metastatic papillary thyroid carcinoma (PTC). This lesion exhibited PTC related nuclear changes and was positive for BRAF1. **B** Low-power image of the 15 mm large deposit of follicular-patterned cells in a separate lateral node, consistent with metastatic follicular thyroid carcinoma (FTC). **C** High-power magnification reveals a microfollicular growth pattern and monotonous appearance, with nuclei clearly lacking PTC associated findings. **D** Schematic overview of the histological and molecular findings. The thyroid lobe is presented in grey, with the red tumor representative of the tall cell variant PTC found in the right lobe, with synchronous spread to central and lateral lymph nodes. These lesions were positive for BRAF1, the V600 specific antibody used to identify *BRAF* V600 mutated cases. The 12 mm minimally invasive FTC is depicted as a blue tumor, with spread to a lateral lymph node. These lesions exhibited an *NRAS* mutation detected by next-generation sequencing. Created using BioRender.com

In the central compartment, 13 lymph nodes were identified, two of them containing metastatic PTC foci. The largest metastatic deposit measured 4 mm, and no extranodal extension was noted. Less than 5% of the cells expressed thyroglobulin. In the lateral compartment, 12 lymph nodes were found, of which one displayed a metastatic PTC focus measuring 1 mm (Fig. 2A). In a separate lymph node, a 15 mm area with micro-follicular patterned cells with central colloid was observed (Fig. 2B). No extranodal extension was observed. The nuclei were small, round and displayed evident nucleoli, but lacked PTC associated nuclear changes (Fig. 2C). Thyroglobulin immunoreactivity was widespread, and the BRAF1 staining was negative. The Ki-67 index was well below 1%.

Given the finding of thyroid-derived, non-PTC tissue in a lateral lymph node, two options were discussed. The finding could represent either ectopic thyroid tissue entrapped within a lymph node, or a lateral lymph node metastasis of the miFTC detected in the right thyroid lobe. However, to our knowledge, lateral aberrant thyroid tissue is a rarity and has only been described in single case reports, and a lateral lymph node metastasis from the 12 mm miFTC was thus favored, although this phenomenon is also acknowledged as an exceptional event. The findings were in contrast to the previous FNAC report suggestive of metastatic PTC, but following re-investigation of the cytological preparations, this former biopsy was considered to derive from the node exhibiting metastatic FTC. To determine the nature of this 15 mm focus and its relation to the miFTC from a molecular standpoint, we extracted DNA from representative paraffin blocks and performed a targeted next-generation sequencing (NGS) analysis using the Oncomine Childhood Cancer Research Assay (Thermo-Fisher Scientific, Waltham, MA, USA), which has been validated for clinical use in our department [6]. The NGS analysis pinpointed a p.Q61R missense *NRAS* mutation in both the primary miFTC as well as the lateral lymph node deposit (Fig. 2D). This variant is an oncogenic mutation in both papillary and follicular thyroid cancer, and given the shared molecular phenotype, the final diagnosis was a 15 mm lateral lymph node metastasis of FTC. We also investigated the primary miFTC for *TERT* promoter mutations using highly sensitive digital droplet PCR (ddPCR), an analysis that has proven superior to conventional Sanger sequencing in identifying these risk mutations in follicular thyroid tumors [7]. However, the miFTC was wildtype at both position C228 and C250 of the *TERT* promoter.

The final histopathology report was signed out as multifocal TCV-PTC, largest focus 30 mm with a synchronous 12 mm miFTC in the ipsilateral lobe, both with lateral lymph node metastases. The pTNM was pT2(m)N1b and pT1bN1b respectively, and surgical margins were negative for both lesions. The patient was post-operatively investigated for a remaining right-sided lymph node enlargement visualized via ultrasound investigation, but the ensuing cytological investigation was negative.

The patient was subsequently discussed at a postoperative multidisciplinary conference. According to the Swedish national guidelines for thyroid cancer, the patient should have been offered 3.7 GBq radioiodine (RAI) as an ablation dose, due to the N1b status. However, the conference suggested the patient to receive 5.4 Gbq RAI, which was based on the tall cell variant found, and the fact that both thyroid cancer components had shown ability to metastasize. The American Thyroid Association (ATA) guidelines would classify the FTC component in our case as “low to intermediate risk” given the current N1b staging, thus suggesting that RAI should be considered, and most often favored due to higher risk of persistent or recurrent disease [1]. Following RAI, an ensuing whole-body scan was negative, and both unstimulated and TSH-stimulated measurements of serum thyroglobulin were < 0.2 µgram/L (ref: < 1 µgram/L for thyroidectomized patients), and serum thyroglobulin antibody levels were < 20 kU/L (ref: < 40 kU/L). The patient is currently prescribed 125 µg levothyroxine daily, with free serum T4 levels at 25 pmol/L (ref: 12–22 pmol/L), T3 at 5.8 pmol/L (3.1–6.8 pmol/L) and TSH at 0.2 mU/L (ref: 0.3–4.2 mU/L). According to the ATA guidelines, our patient thus exhibits excellent response [1]. She is alive and well, although monitored by our endocrinologists.

## Discussion and conclusions

Consulting the literature, several case reports and case series describing synchronous manifestation of PTC and FTC have been found, whereas reports of synchronous lateral lymph node engagement are virtually absent [8]. Regional lymph nodes metastases are reported to occur in less than 10% of FTCs and Hürthle cell carcinomas [2, 9–11]. Even fewer cases are described in which the engaged lymph nodes are located outside of the head and neck area [12]. The reason why our index patient exhibited co-occurring PTC and FTC lymph node metastases is not known, and particularly puzzling given the fact that the primary FTC was a small tumor with limited capsular invasion and a rather low Ki-67 proliferation count. Moreover, this patient was not immuno-compromised in any way, thus arguing against a faulty immune response.

In terms of prognostication via molecular genetic analyses, the index patient’s FTC was *TERT* promoter wildtype at positions C228 and C250, thus arguing against poorer outcome [13–15]. Moreover, we did not find any somatic variants in the *DICER1* gene, a recurrently mutated gene in WDTCs and poorly differentiated thyroid carcinomas arising in the adolescent population [6, 16, 17]. The observed Q61R *NRAS* mutation is a well-established driver of follicular thyroid tumors and occurs in subsets of follicular variant PTCs, thus proving that the lateral lymph node tissue was tumorous rather than an ectopically located inclusion of benign thyroid epithelia [18]. Our case mirrors previous publications regarding the molecular profile of synchronous PTC and FTC, describing somatic Q61R *NRAS* mutation in an FTC developing alongside a *BRAF* mutated PTC [8]. Although the somatic status of these driver mutations is acknowledged, we did not interrogate germline DNA for any type of susceptibility gene variant, which is a limitation to the report.

From a therapeutic perspective, the most aggressive neoplasm should probably guide the treatment when the patient presents with two synchronous thyroid malignancies [19]. In our case, this was not easily defined, as the PTC was larger than the co-occurring FTC, and also of a high-risk subtype (tall cell variant). Even so, the manifestation of metastatic FTC should mandate careful considerations, as the lack of knowledge regarding locally metastatic cases is substantial. ATA has proposed a risk stratification model in which the AJCC/TNM staging system is considered, ranging from “low risk” to “high risk”, in which the indication for postoperative RAI treatment is debatable for low-risk cases while recommended for high risk tumors with either a T4 status and/or known distant metastases [1]. Smaller tumors without extrathyroidal extension (stage T1-3) but with evident lateral lymph node metastases (stage N1b) are designated as “low to intermediate risk” by ATA, and the potential use of RAI in this group (as well as in our case) is debatable in terms of improvement of disease-free survival. Even so, RAI is generally favored as the risk of recurrent disease is not neglectable [1]. As the PTC component in our case was a *BRAF* mutated tall cell variant, this would count as an intermediate risk tumor according to ATA guidelines, therefore further supporting the administration of RAI to this specific patient [1]. It should also be stressed that algorithms exist in which the authors suggest a dynamic risk assessment for differentiated thyroid cancer treated with surgery and RAI, in which the assessment of response to initial therapy should be considered during the 2 first years of follow-up [20]. For our patient with limited follow-up, such a dynamic risk evaluation would need additional observation time, but since no clinical, biochemical or structural evidence of disease exist post-surgically as of today, the risk of disease recurrence is believed to be low.

We conclude that lateral lymph node metastases from FTCs are exceedingly rare, and demonstrate the value of NGS analyses to pinpoint the metastatic nature of the lesion and to rule out intra-nodal, ectopic thyroid tissue. Moreover, the lack of available reports in which synchronous lymph node metastases of PTC and FTC are described demand a personalized approach to the clinical management which should be restricted to tertiary thyroid centers.

## Data Availability

All data generated or analyzed during this study are included in this published article.

## Abbreviations

AJCC: American Joint Committee on Cancer
ATA: American Thyroid Association
BRAF: V-Raf Murine Sarcoma Viral Oncogene Homolog B1
BRAF1: V600 mutation-specific BRAF antibody
ddPCR: Digital droplet polymerase chain reaction
FNAC: Fine-needle aspiration cytology
FTC: Follicular thyroid carcinoma
GBq: Giga-Becquerel
miFTC: Minimally invasive follicular thyroid carcinoma
NRAS: Neuroblastoma RAS Viral Oncogene Homolog
PTC: Papillary thyroid carcinoma
pTNM: Pathological tumor, nodes, metastasis staging
RAI: Radioiodine
TCV-PTC: Tall cell variant of papillary thyroid carcinoma
TERT: Telomerase reverse transcriptase
TTF1: Thyroid transcription factor 1

## References

[1] Haugen BR, Alexander EK, Bible KC, Doherty GM, Mandel SJ, Nikiforov YE (2016). 2015 American Thyroid Association Management Guidelines for Adult Patients with Thyroid Nodules and Differentiated Thyroid Cancer: The American Thyroid Association Guidelines Task Force on Thyroid Nodules and Differentiated Thyroid Cancer. Thyroid.

[2] Zaydfudim V, Feurer ID, Griffin MR, Phay JE (2008). The impact of lymph node involvement on survival in patients with papillary and follicular thyroid carcinoma. Surg.

[3] Barbieri A, Prasad ML, Gilani SM (2020). Thyroid tissue outside the thyroid gland: Differential diagnosis and associated diagnostic challenges. Ann Diagn Pathol.

[4] Nakayama DK (2018). Lateral Ectopic Thyroid in a Teenaged Girl. Am Surg.

[5] Baek MK, Kim DY, Woo JH (2015). A case of pediatric ectopic thyroid in lateral lymph nodes. J Pediatr Endocrinol Metab.

[6] Juhlin CC, Stenman A, Zedenius J (2021). Macrofollicular variant follicular thyroid tumors are DICER1 mutated and exhibit distinct histological features. Histopathol.

[7] Hysek M, Jatta K, Hellgren LS, Stenman A, Larsson C, Zedenius J (2021). Spatial Distribution Patterns of Clinically Relevant TERT Promoter Mutations in Follicular Thyroid Tumors of Uncertain Malignant Potential: Advantages of the Digital Droplet PCR Technique. J Mol Diagn.

[8] Cracolici V, Mujacic I, Kadri S, Alikhan M, Niu N, Segal JP (2018). Synchronous and Metastatic Papillary and Follicular Thyroid Carcinomas with Unique Molecular Signatures. Endocr Pathol.

[9] Pisanu A, Deplano D, Pili M, Uccheddu A (2011). Larger tumor size predicts nodal involvement in patients with follicular thyroid carcinoma. Tumori.

[10] Lin JD, Liou MJ, Chao TC, Weng HF, Ho YS (1999). Prognostic variables of papillary and follicular thyroid carcinoma patients with lymph node metastases and without distant metastases. Endocr Relat Cancer.

[11] Coca-Pelaz A, Rodrigo JP, Shah JP, Sanabria A, Al Ghuzlan A, Silver CE (2021). Hürthle Cell Carcinoma of the Thyroid Gland: Systematic Review and Meta-analysis. Adv Ther.

[12] Li T, Ma Z, Lu C, Zhou Q, Feng Z, Wu X (2019). Chest wall lymph node metastasis from follicular thyroid carcinoma: a rare case report. Diagn Pathol.

[13] Bournaud C, Descotes F, Decaussin-Petrucci M, Berthiller J, de la Fouchardière C, Giraudet A-L (2019). TERT promoter mutations identify a high-risk group in metastasis-free advanced thyroid carcinoma. Eur J Cancer.

[14] Liu T, Wang N, Cao J, Sofiadis A, Dinets A, Zedenius J (2014). The age- and shorter telomere-dependent TERT promoter mutation in follicular thyroid cell-derived carcinomas. Oncogene.

[15] Paulsson JO, Mu N, Shabo I, Wang N, Zedenius J, Larsson C (2018). TERT aberrancies: a screening tool for malignancy in follicular thyroid tumours. Endocr Relat Cancer.

[16] Chernock RD, Rivera B, Borrelli N, Hill DA, Fahiminiya S, Shah T (2020). Poorly differentiated thyroid carcinoma of childhood and adolescence: a distinct entity characterized by DICER1 mutations. Mod Pathol.

[17] Wasserman JD, Sabbaghian N, Fahiminiya S, Chami R, Mete O, Acker M (2018). DICER1 Mutations Are Frequent in Adolescent-Onset Papillary Thyroid Carcinoma. J Clin Endocrinol Metab.

[18] Romei C, Elisei R (2021). A Narrative Review of Genetic Alterations in Primary Thyroid Epithelial Cancer. Int J Mol Sci.

[19] Ryan N, Walkden G, Lazic D, Tierney P (2015). Collision tumors of the thyroid: A case report and review of the literature. Head Neck.

[20] Tuttle RM, Tala H, Shah J, Leboeuf R, Ghossein R, Gonen M (2010). Estimating risk of recurrence in differentiated thyroid cancer after total thyroidectomy and radioactive iodine remnant ablation: using response to therapy variables to modify the initial risk estimates predicted by the new American Thyroid Association staging system. Thyroid.

